# Field Evaluation of Recombinant Antigen ELISA in Detecting Zoonotic Schistosome Infection Among Water Buffaloes in Endemic Municipalities in the Philippines

**DOI:** 10.3389/fvets.2020.592783

**Published:** 2020-10-15

**Authors:** Jose Ma. M. Angeles, Yasuyuki Goto, Masashi Kirinoki, Elena A. Villacorte, Kharleezelle J. Moendeg, Pilarita T. Rivera, Yuichi Chigusa, Shin-ichiro Kawazu

**Affiliations:** ^1^Department of Parasitology, College of Public Health, University of the Philippines, Manila, Philippines; ^2^National Research Center for Protozoan Diseases, Obihiro University of Agriculture and Veterinary Medicine, Obihiro, Japan; ^3^Laboratory of Molecular Immunology, Department of Animal Resource Sciences, Graduate School of Agricultural and Life Sciences, The University of Tokyo, Tokyo, Japan; ^4^Laboratory of Tropical Medicine and Parasitology, Dokkyo Medical University, Tochigi, Japan; ^5^Department of Biology, School of Science and Engineering, Ateneo de Manila University, Quezon City, Philippines

**Keywords:** zoonotic schistosomiasis, *Schistosoma japonicum*, water buffaloes, SjTPx-1, Sj1TR, diagnosis

## Abstract

In this study, we investigated the use of recombinant antigens thioredoxin peroxidase-1 (rSjTPx-1) and tandem repeat rSj1TR in evaluating the antibody positivity rates of *Schistosoma japonicum* infection among water buffaloes from four endemic areas in the Philippines, two municipalities with high endemicity (Calatrava, Negros Occidental and Catarman, Northern Samar) and two municipalities nearing elimination with no cases of human schistosomiasis (Talibon and Trinidad, Bohol). These recombinant antigen ELISA assays were compared with other diagnostic tests including SEA-ELISA, FECT, and fecal-based PCR. Results showed that rSj1TR-ELISA has the highest agreement with PCR in all study areas. Furthermore, significant positivity rates among water buffaloes were seen in Talibon and Trinidad, indicating that water buffaloes are maintaining the schistosome parasites in transmission areas even in the absence of human infection. Hence, serological assay using a more sensitive and specific rSj1TR-ELISA can be used for animal surveillance to prevent emergence and re-emergence of human schistosomiasis.

## Introduction

Water buffaloes are an important reservoir for *Schistosoma japonicum* in schistosomiasis-endemic countries including China and the Philippines. Because of their continuous exposure to the parasite in transmission areas, it was suggested that water buffaloes should be considered as the sentinel animal in the surveillance of zoonotic schistosomiasis (1). Interruption of transmission can be confirmed and will help achieve possible elimination of schistosomiasis in many areas where the prevalence has been reduced to near elimination levels. However, there are still no available standard tests on detecting the schistosome infection in these animals.

Molecular diagnostic tests such as real-time polymerase chain reaction (qPCR) have been used in several researches to determine prevalence of this parasitic disease in water buffaloes and showed high sensitivity and specificity (2). In our previous study, we have used a conventional PCR targeting the NAD 1 gene of *S. japonicum* for water buffaloes as a reference standard for the serological evaluation of recombinant antigens (3). Using fecal samples spiked with *S. japonicum* eggs, it has a detection limit of one schistosome egg proving its high sensitivity (unpublished data). However, these tests require expertise, costly reagents and equipment that may not be available in remote, endemic areas. On the other hand, a tedious parasitological technique called Danish Bilharziasis Laboratory method (DBL) employing several sieves in catching the schistosome eggs has been recommended as an alternative means of diagnosis in animals (4). This method is time-consuming, laborious and cannot be used in large surveys.

Zoonotic schistosomiasis is a chronic parasitic disease and in water buffaloes where exposure to the schistosome parasite in transmission sites is constant and continuous, antibody detection seems to be the most appropriate diagnostic means. However, serological tests using crude antigens cause cross-reactions that might lead to false-positives and misdiagnosis, especially in regions where more than one trematode parasite is endemic. The use of parasite specific recombinant proteins therefore will increase the precision and accuracy of serological diagnosis. Moreover, several recombinant antigens detecting anti-schistosome antibodies have shown promising results in previous studies (3, 5). Prior to this study, we have already identified recombinant thioredoxin peroxidase-1 (rSjTPx-1) and the tandem repeat protein rSj1TR as antigens with good diagnostic potentials for water buffaloes in *S. japonicum* infection as compared to the crude soluble egg antigen (SEA) using the enzyme-linked immunosorbent assay (ELISA) (3). SjTPx-1 and Sj1TR also showed no cross-reaction when tested against samples from goats infected with *Fasciola hepatica* (3). Field evaluation of these recombinant antigen ELISA assays is needed to know the potential diagnostic applicability in the surveillance of zoonotic schistosomiasis among water buffaloes. In this study, we compared the ELISA utilizing rSjTPx-1 and rSj1TR with the parasitological formol-ether concentration technique (FECT) and fecal sample-based PCR in evaluating positivity rates of *S. japonicum* infection among water buffaloes in several endemic municipalities in the Philippines.

## Materials and Methods

### Study Area and Sampling

Serum and fecal samples were collected in the period of March 2012 to April 2014 from water buffaloes in schistosomiasis-endemic provinces in the Philippines namely Calatrava, Negros Occidental (*n* = 59), Talibon (*n* = 28) and Trinidad (*n* = 32), Bohol and Catarman, Northern Samar (*n* = 49). Human prevalence were recorded in Calatrava to be at least 5% according to a focal survey (6) and in Northern Samar with 2.5% based on a nationwide survey (7) using Kato-Katz technique. No cases of human schistosomiasis have been reported in Talibon and Trinidad since 2012 based on municipal health records (unpublished data).

### Fecal Sample Analysis

Fecal samples were collected through intrarectal means from the water buffaloes, placed in code-labeled cups and stored with 10% neutralized formalin until use. FECT was performed on the fecal samples based on previous protocols (8). Each fecal sediment from FECT was divided for microscopy and DNA extraction. Three fecal smears were examined microscopically for parasite eggs per sample. On the other hand, DNA extraction was processed using QIAamp DNA Stool Mini Kit (QIAGEN Inc., Valencia, CA, USA) based on the manufacturer's protocol.

**Figure 1:**
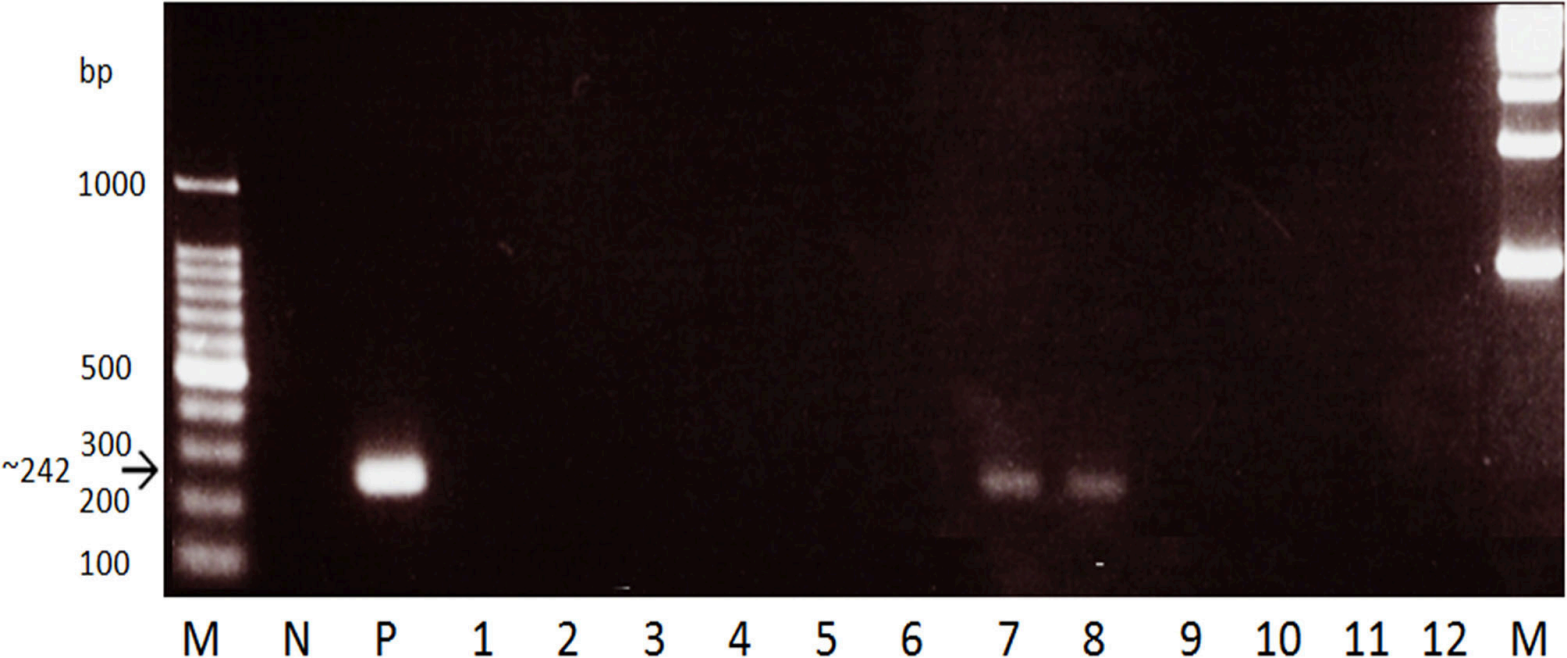
Gel electrophoresis of the stool PCR for water buffaloes targeting the *S. japonicum* mitochondrial *cox2-nad6* gene. *M*, marker. *N*, negative control (stool DNA from non-endemic cattle). *P*, positive control (*S. japonicum* adult DNA template). *Lanes 1-12*, Water buffalo samples. A 242 bp band was seen in positive samples while none in negative.

Fecal sample-based PCR amplifying a 242 bp mitochondrial marker from a region at position 4,961 within the *cox2* gene to position 7,180 within the *nad6* gene was used on the water buffalo fecal samples (9). The primer set 5′-GCCGTTACGCTTAGAGCG-3′ forward primer and 5′-CATCCAAGCCGATTACCC-3′ reverse primer was utilized. A 20 μl reaction contained 2 μl of 10 × PCR buffer, 0.6 μl of 1.5 mM MgCl_2_, 1.6 μl of 2.5 mM dNTP, 0.4 μl of each 20 pmol/μl primer, 0.2 μl of 5 U/μl *Taq* DNA polymerase (Takara, Otsu, Japan) and 2 μl of template from the fecal DNA isolation. The cycling program used was as follows: initial denaturation at 95°C for 15 min, followed by 45 cycles of 94°C for 30 s denaturation, 63°C for 1 min annealing, 72°C for 90 s extension, and a final extension of 72°C for 10 min. After performing the PCR using Veriti 96 Well Thermal Cycler (Applied Biosystems, Carlsbad, CA, USA), the PCR products were separated by electrophoresis in 1.5% agarose gel and sizes were resolved using a 100 bp DNA ladder (Invitrogen, Tokyo, Japan). The bands were then visualized by ethidium bromide staining. PCR reactions were performed in triplicates for every fecal sample and a sample is regarded as positive when at least one reaction showed the 242 bp band in the visualized gel (Figure 1).

### ELISA

For the ELISA, SEA and the recombinant proteins rSjTPx-1 and rSj1TR were prepared and used according to our previous paper (4). In brief, 96-well microplates (Nunc Maxisorp, Thermo Fisher, Rockland, IL) were sensitized separately with SEA (1 μg/well) or each of the recombinant proteins (200 ng/well). The test sera (0.1 ml) were diluted 200-fold in Tween 20-phosphate buffered solution with 1% bovine serum albumin whereas the secondary antibody (0.1 ml) was diluted in 10,000-fold. Secondary antibody horseradish peroxidase-conjugated Protein G (Rockland Inc., Gilbertsville, PA, USA) and the substrate 3,3',5,5'-tetramethylbenzidine (KPL, Gaithersburg, MD, USA) were used. Optical density (OD) at 450 nm was measured using a microplate reader (MTP-500, Corona Electric, Tokyo, Japan). All the tests were done in triplicates and data represent mean values. A sample was considered positive when the mean absorbance value of each sample was higher than the cut-off value. The cut-off values were calculated as mean plus three standard deviation of the absorbance values from negative samples collected from municipalities in the Philippines known to be non-endemic for schistosomiasis (3).

### Statistical Analysis

Diagnostic parameters including the sensitivity, specificity, positive predictive values, negative predictive values, and accuracy were calculated using the field samples. Kappa statistics was used to qualitatively measure the magnitude of the agreement between the tests with fecal sample-based PCR as the reference test (10). A kappa value near 1 was interpreted as having a perfect agreement whereas a value near 0 as just having a less than chance agreement (11). All the statistical analyses done were calculated using the statistics software GraphPad (San Diego, CA, USA).

### Study Ethics

All the owners of the water buffaloes were informed about the background of this study and signed consent forms for the use of their water buffaloes in this study. This study was done according to ethical guidelines for the epidemiological use of animal samples provided by Obihiro University of Agriculture and Veterinary Medicine (Permit No. 23-153 and No. 26-31).

## Results

As shown in Table 1, positivity rates for schistosome infection are high in the highly endemic areas of Calatrava (x = 27.46) and Catarman (x = 45.72). However, there are marked differences in positivity rates in these two municipalities using the sensitive fecal sample-based PCR (49.15, 40.82%) and microscopy (6.78, 20.41%). Furthermore, microscopy detected the lowest number of positive samples among the tests in all the endemic areas examined. While all the microscopy positive samples tested positive for PCR, not all those which tested positive for the PCR were positive by microscopy showing that agreements between the two tests are very low in all these areas except in Talibon. Also, marked positivity rates were seen in Talibon (x = 15.71) and Trinidad (x = 20.63) where no human cases of schistosomiasis were reported for 2 years prior to the collection.

**Table 1:** Positivity rate of schistosome infection among the water buffaloes in selected endemic municipalities using different diagnostic tests.

**Diagnostic tests**	**Calatrava** ***n*** **=** **59**		**Catarman** ***n*** **=** **49**		**Talibon** ***n*** **=** **28**		**Trinidad** ***n*** **=** **32**	
	**No. of positives**	**Positivity rate (%)**	**No. of positives**	**Positivity rate (%)**	**No. of positives**	**Positivity rate (%)**	**No. of positives**	**Positivity rate (%)**
Microscopy	4	6.78	10	20.41	2	7.14	2	6.25
SEA-ELISA	15	25.42	34	69.39	11	39.29	14	43.75
Sj1TR-ELISA	21	35.59	19	38.78	3	10.71	6	18.75
SjTPx-1-ELISA	12	20.34	29	59.18	4	14.29	7	21.88
Stool PCR	29	49.15	20	40.82	2	7.14	4	12.50
Mean positivity rates (x)		27.46		45.72		15.71		20.63

**Figure 2:**
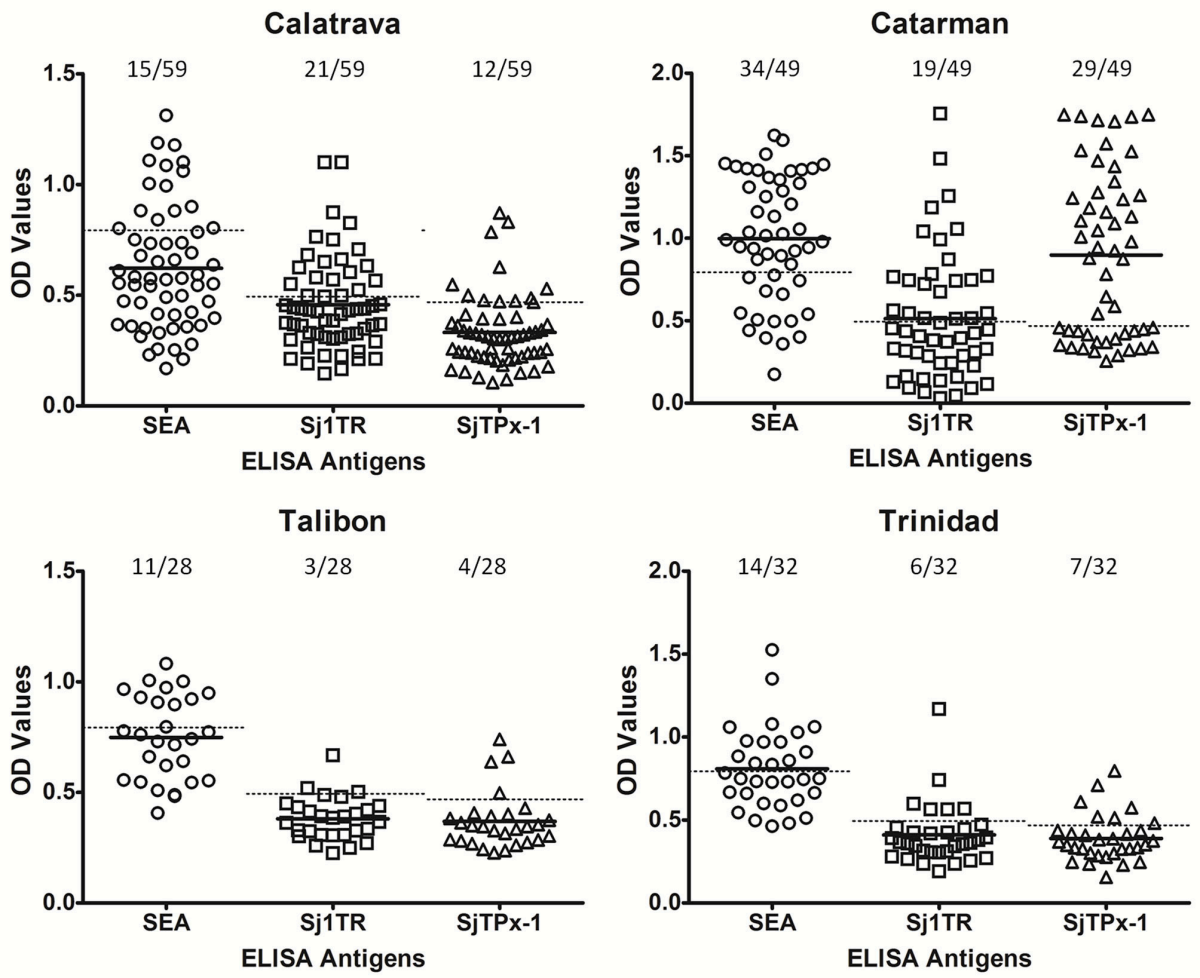
ELISA results of the water buffaloes from selected schistosomiasis-endemic areas. Cut-off values were shown as dotted lines whereas mean OD values as solid lines.

rSjTPx-1 and rSj1TR were proven to have good diagnostic potentials in our previous studies for water buffalo diagnosis (3). rSjTPx-1 also showed remarkable sensitivity and specificity in humans (12) and dogs (13). These recombinant antigens were used in this study in the ELISA format together with the crude SEA for comparison. The positivity rate on all the tested antigens showed correlation with the infection prevalence in different geographical areas; in the highly endemic municipalities, the antigens showed high positivity rates and vice versa (Table 1). In terms of serological reactivity, SEA detected the higher number of positive samples than the other two defined antigens in all these endemic areas except in Calatrava (Figure 2).

In our previous evaluation of recombinant diagnostic antigens for water buffaloes, rSjTPx-1 and rSj1TR showed 82.61% sensitivity, 96.67% specificity, and 78.26% sensitivity, 93.33% specificity, respectively as compared to SEA having 97.14% sensitivity and 71.76% specificity (3). However, field sensitivity and specificity of rSj1TR-ELISA were shown to be lower in this study with 68.52 and 87.61%, respectively (Table 2). The highest among the ELISA was shown in rSj1TR-ELISA with 83.33% sensitivity and 97.35% specificity. Accuracy was also calculated with 92.81% for rSj1TR and 81.44% for rSjTPx-1.

**Table 2:** Statistical analysis of the diagnostic potentials of ELISA using SEA and recombinant antigens rSjTPx-1 and rSj1TR*.

	**SEA-ELISA**	**rSj1TR-ELISA**	**rSjTPx-1-ELISA**
Sensitivity (%)	70.37	83.33	68.52
Specificity (%)	69.03	97.35	87.61
Positive predictive value (%)	52.05	93.75	72.55
Negative predictive value (%)	82.98	92.44	85.34
Accuracy (%)	69.46	92.81	81.44

* *All the diagnostic parameters used were calculated using the statistics software GraphPad. Stool PCR served as the reference test*.

Furthermore, rSj1TR showed the highest agreement with fecal sample-based PCR in all the examined municipalities giving an overall kappa value of 0.818, with a clear contrast to SEA having the lowest agreement with PCR with an overall kappa value of 0.292 (Table 3).

**Table 3:** Statistical agreement of the diagnostic tests in selected schistosomiasis-endemic municipalities.

**Endemic municipalities**	Kappa values*			
	**Microscopy**	**SEA-ELISA**	**Sj1TR-ELISA**	**SjTPx-1-ELISA**
Catarman	0.542	0.314	0.914	0.645
Calatrava	0.140	0.316	0.727	0.281
Talibon	1.000	0.213	0.781	0.632
Trinidad	0.636	0.310	0.765	0.676
Total	0.396	0.292	0.818	0.520

* *Kappa values were calculated using the statistics software GraphPad. Stool PCR served as the reference test*.

## Discussion

In this study, we have compared rSjTPx-1 ELISA and rSj1TR ELISA with SEA-ELISA, FECT and PCR in determining the positivity rates of the water buffaloes in four endemic areas in the Philippines. The highest positivity rate was seen in SEA-ELISA, but it was known that crude antigens like SEA have low specificity and could cause cross-reaction with other parasitic diseases in both humans (12) and animals (3, 13). In addition, SEA-ELISA cannot distinguish past and present infection. This is a clear contrast with rSjTPx-1 and rSj1TR which brought negative results in human sera collected 1 year after praziquantel treatment in our previous study (12) suggesting that these recombinant antigens are useful in detecting active human schistosomiasis. However, this may not be the case in water buffaloes that are known to experience the self-cure phenomenon (14) as well as a steady exposure to the parasite that might greatly affect their immunological profile. A more intensive study should therefore be done to clearly validate the use of these recombinant antigens in detecting active cases of schistosome infection in water buffaloes.

Our results showed microscopy to have the lowest positivity rates among the water buffaloes in the study areas. On the other hand, all the schistosome egg positive samples were also positive with fecal sample-based PCR thus verifying the sensitivity of the PCR in detecting schistosomiasis even in areas where infection intensity is low. Among the recombinant antigens, rSj1TR showed the highest field sensitivity, specificity and agreement with the PCR suggesting its better suitability as a diagnostic antigen in the water buffalo surveillance than the other antigens used. Interestingly, there were samples tested positive for the recombinant antigen ELISA but not in PCR. This could be explained by individuals who are not excreting eggs seen at the early stages of the disease or those with very low egg burden (15) and with the large day-to-day variation in the egg output (16, 17). This has emphasized the advantage of antibody testing over molecular techniques in the diagnosis of schistosome infection. For ease of use, rSj1TR should be tested in future studies in lateral flow format to develop a sensitive and specific rapid diagnostic test for zoonotic schistosomiasis.

Our results indicate a marked positivity among the water buffaloes tested in near elimination areas of Talibon and Trinidad where no human cases have been recorded since 2012. Presence of the schistosome parasite in the bovine hosts poses a threat in the human schistosomiasis elimination. As seen in the previously controlled areas of Anhui and Sichuan provinces of China, re-emergence of schistosomiasis in humans was significantly attributed to the high prevalence of schistosome infection among cows and water buffaloes (18, 19). This threat can possibly be avoided by setting up a sensitive animal surveillance system that can detect infection in animals for prompt intervention. Hence, animals serving as sources of infection can be treated properly preventing any further contamination of the environment with the schistosome eggs that can start transmission anew.

Also, the presence of the parasites in the water buffaloes suggest the underestimation of the human prevalence in the surveys done using the less sensitive Kato-Katz technique (20). The intensive mass drug administration done in the endemic areas have resulted to infected individuals to manifest low intensity of infection (21). This greatly affects the diagnostic performance of stool based diagnostic assays such as Kato-Katz and indicate the need for more sensitive diagnostic tests to be used in such conditions. In addition, our results showed that the positivity rates in the rSj1TR-ELISA is directly proportional with the level of human prevalence in the four municipalities. This is also true for rSjTPx-1-ELISA except for the samples from Calatrava, Negros Occidental. As SjTPx-1 is only one of the detoxifying agents against hydrogen peroxide in helminths (22), its expression levels for each geographical isolate should be analyzed. This might affect its antigenicity and results to low antibody levels in some groups of water buffaloes as seen in the Calatrava samples.

The possibility of genetic and strain differences between human and animal schistosomes cannot be discounted and should be further explored through molecular means. Nonetheless, our results showing positivity for schistosome parasites among the examined water buffaloes in Talibon and Trinidad still prove that in near elimination areas, human cases may be zero but animal infections may constantly persist, and continue to perpetuate the transmission of schistosomiasis. Hence, animal surveillance using a more sensitive and specific test should be done as means of regularly monitoring animal infection. And in such cases should the water buffaloes be found positive with the schistosome parasites as seen in the municipalities of Talibon and Trinidad in this study, investigation of the transmission sites including the examination of the snail intermediate hosts must be done to see the entire epidemiological picture of the disease in the area. Based on this information, the local health and veterinary agencies can come up with the appropriate control measures to be pursued such as selective bovine chemotherapy (23, 24) and isolation of the infected water buffaloes to possibly stop transmission.

## Conclusion

rSj1TR has shown to be a better ELISA antigen than rSjTPx-1 in the field evaluation for schistosomiasis detection among water buffaloes. Results showed that rSjTPx-1 ELISA has higher agreement with fecal sample-based PCR than microscopy. Serological tests therefore using recombinant antigens may be useful for the detection of schistosome infection in water buffaloes, if fecal sample-based PCR will not be feasible and available. They should be included in the animal surveillance of the national schistosomiasis control program to ensure elimination and prevent emergence and re-emergence of this parasitic disease.

## Data Availability Statement

The raw data supporting the conclusions of this article will be made available by the authors, without undue reservation.

## Ethics Statement

The animal study was reviewed and approved by Obihiro University of Agriculture and Veterinary Medicine. Written informed consent was obtained from the owners for the participation of their animals in this study.

## Author Contributions

JA, YG, and S-iK conceived and designed the study. JA, EV, KM, PR, and S-iK collected the samples. JA performed the antigen evaluation and analysis. YG, MK, and YC contributed in the data analysis. JA and S-iK prepared the initial draft of the manuscript. All authors read and approved the final manuscript.

## Conflict of Interest

The authors declare that the research was conducted in the absence of any commercial or financial relationships that could be construed as a potential conflict of interest.
